# Supplementary material for: Novel RNA Viruses Discovered in Weeds in Rice Fields
Source: Viruses. 2022 Nov 10;14(11):2489. doi: 10.3390/v14112489 (PMC9717734; doi:10.3390/v14112489)
Supplement: Supplementary file 1 [file viruses-14-02489-s001.zip › Supplementary Material.pdf]

Supplementary Figure

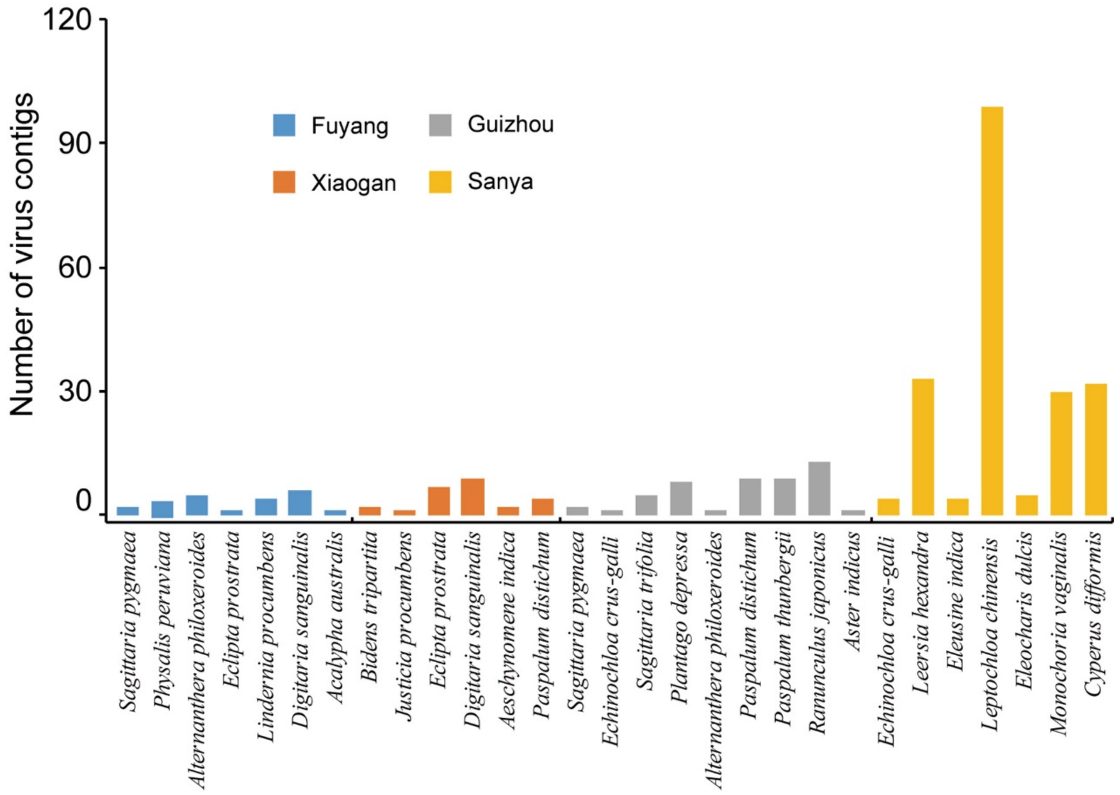

Figure S1. Overview of viral contigs in each weed species. The colors of the bars indicate the sampling locations of the viral genomes. The name of each weed species is shown under each bar.

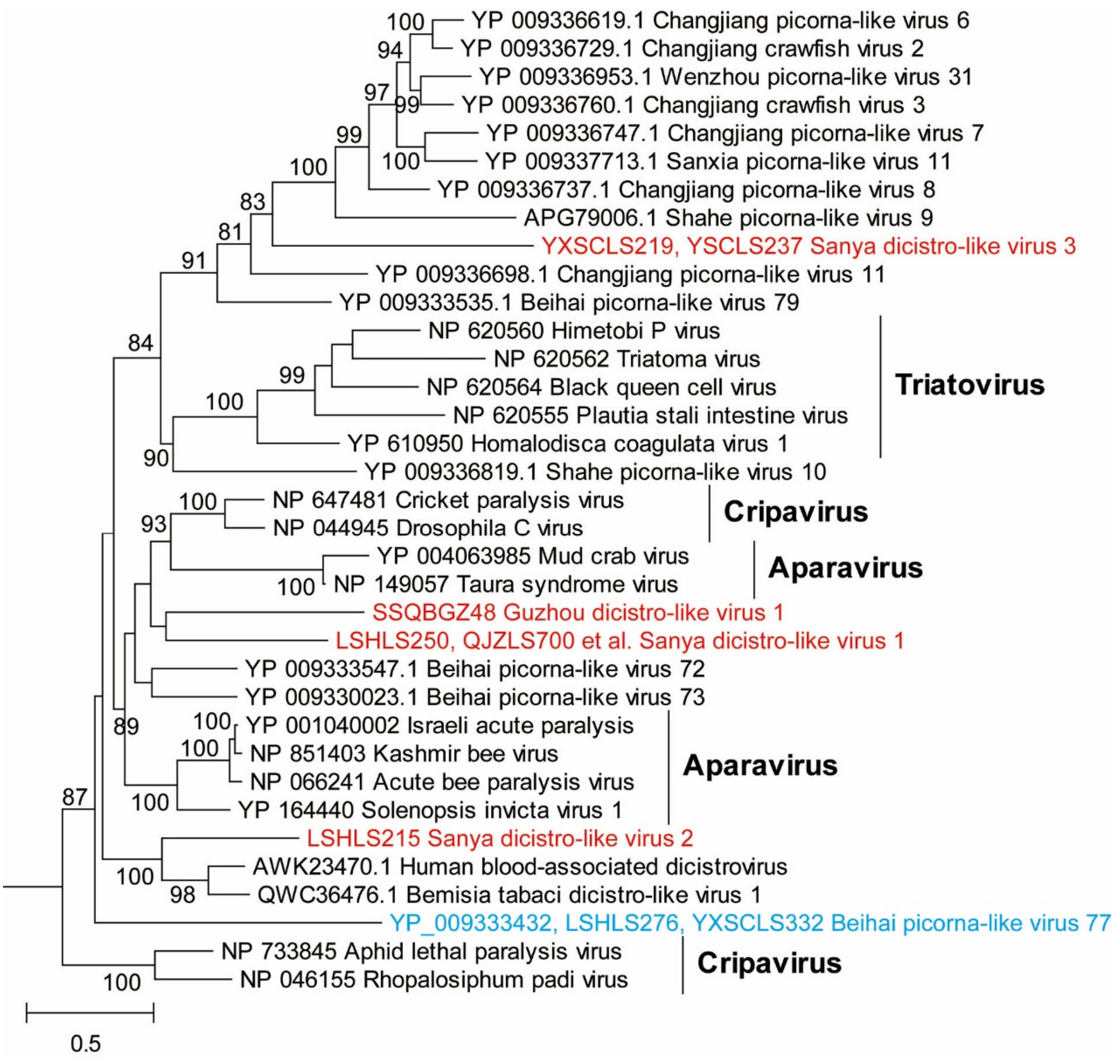

Figure S2. ML phylogenetic analysis of the family *Dicistroviridae*. Phylogenetic trees were constructed using the aligned sequences of RdRp. The names of new viruses discovered in this study are marked in red, the names of known viruses discovered in this study are marked in blue, the reference virus serial numbers and names are marked in black. The genera of viruses (and branches in minimized trees) in this study are marked in bold.

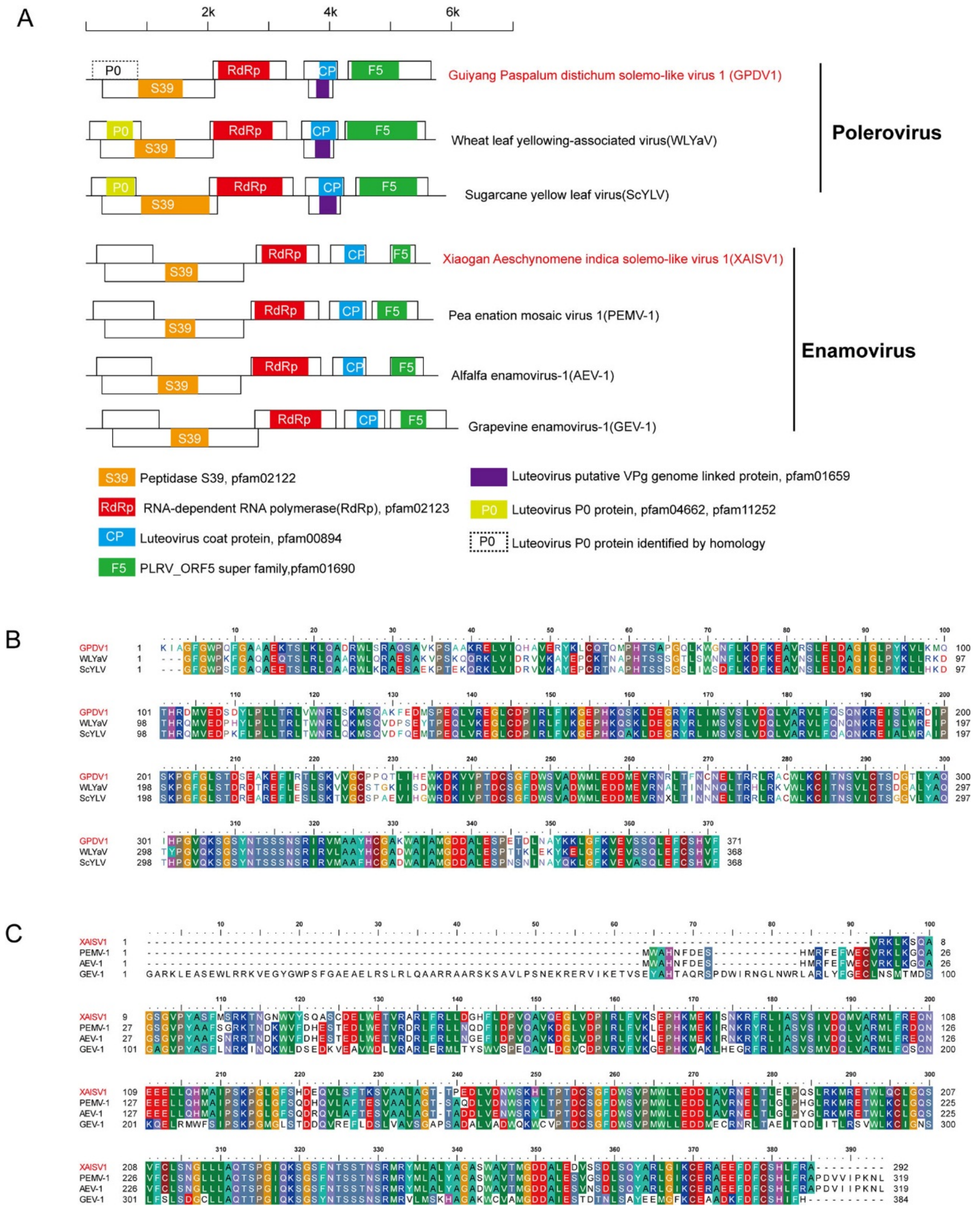

Figure S3. Sequence properties of new solemoviruses identified in this study. (A) Genome structure of Guiyang paspalum paspaloides solemo-like virus 1 (GPDV1) and Xiaogan aeschynomene indica solemo-like virus 1 (XAISV1) with their closest related viruses. The names of new viruses in this study are marked in red; the names of the closest relatives are marked in black. The families of viruses are marked on the right side of the genome structure. (B) Amino acid sequence alignment of solemovirus RdRp reveals conserved regions of GPDV1, wheat leaf yellowing-associated virus (NC_035451) and Sugarcane yellow leaf virus (NC_000874). (C) Amino acid sequence alignment of solemovirus RdRp reveals conserved regions of XAISV1, alfalfa enamovirus-1 (AEV-1) (GenBank no. NC_029993), pea enation mosaic virus 1 (PEMV-1) (GenBank no. NC_003629) and Grapevine enamovirus-1 (GEV-1)

(GenBank no. NC_034836).

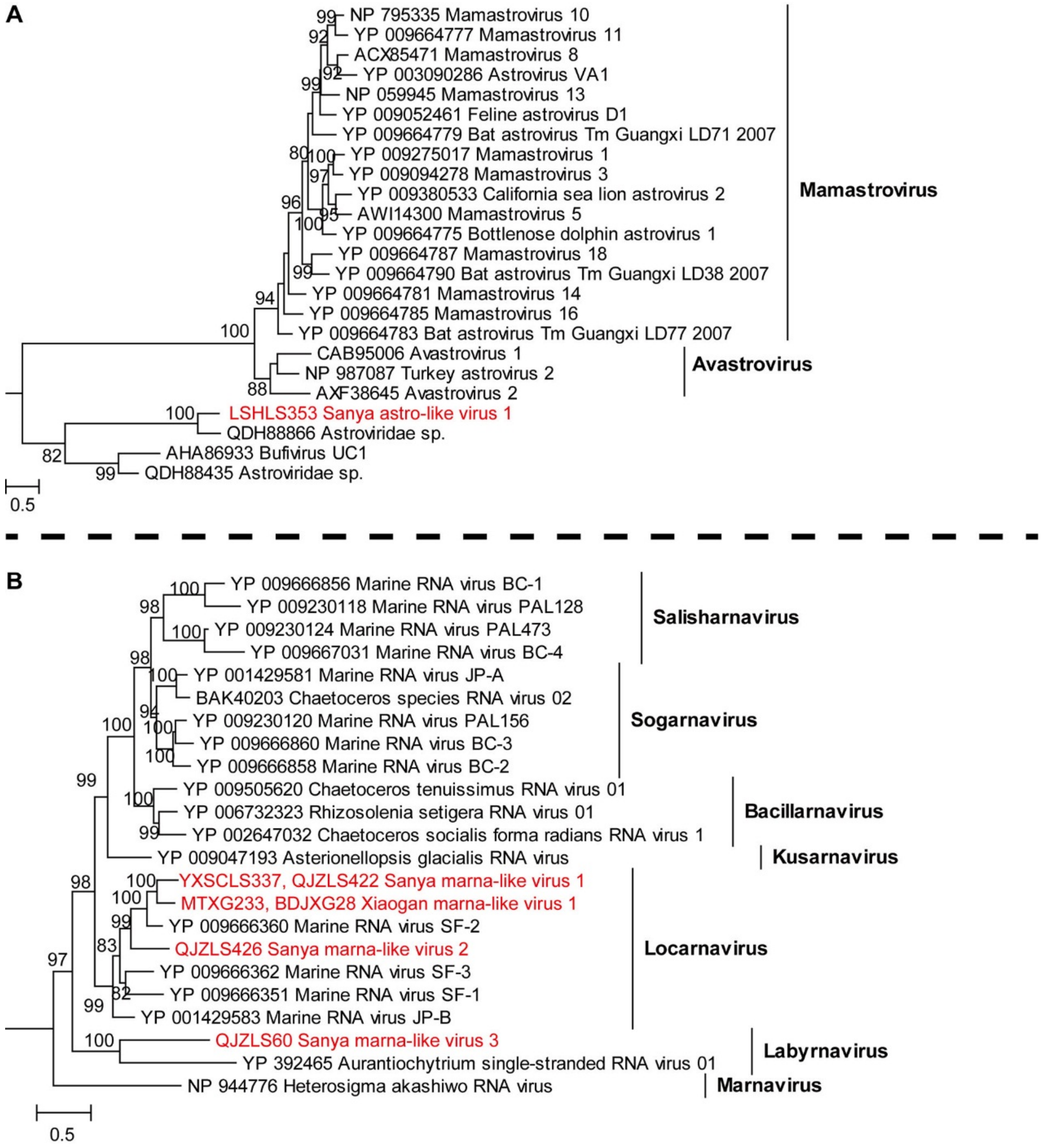

Figure S4. ML phylogenetic analysis of the family *Astroviridae* **(A)** and *Marnaviridae* **(B)**. Phylogenetic trees were constructed using the aligned sequences of RdRp. The names of new viruses discovered in this study are marked in red, the reference virus serial numbers and names are marked in black. The genera of viruses (and branches in minimized trees) in this study are marked in bold.

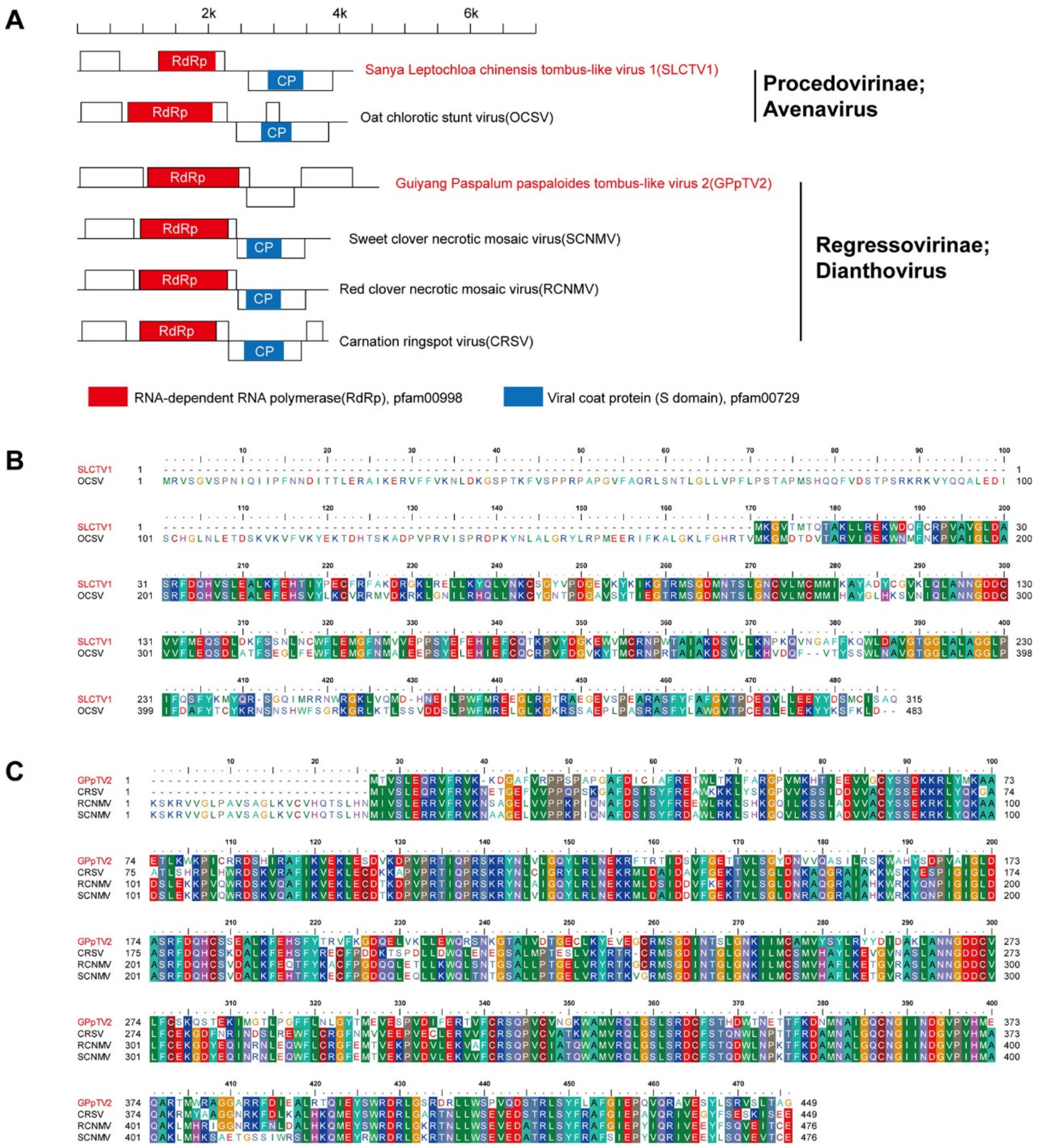

Figure S5. Sequence properties of new tombusviruses identified in this study. **(A)** Genome structure of Sanya Leptochloa chinensis tombus-like virus 1 (SLCTV1) and Guiyang Paspalum paspaloides tombus-like virus 2 (GPpTV2) with their closest relatives. The names of new viruses in this study are marked in red; the names of the closest relatives are marked in black. The families of viruses are marked on the right side of the genome structure. **(B)** Amino acid sequence alignment of tombusvirus RdRp reveals conserved structures of SLCTV1 and Oat chlorotic stunt virus (OCSV) (GenBank no. NC_003633). **(C)** Amino acid sequence alignment of tombusvirus RdRp reveals conserved regions of GPpTV2, Carnation ringspot virus (CRSV) (GenBank no. NC_003530), Red clover necrotic mosaic virus (RCNMV) (GenBank no. NC_003756), and Sweet clover necrotic mosaic virus (SCNMV) (GenBank no. NC_003806).

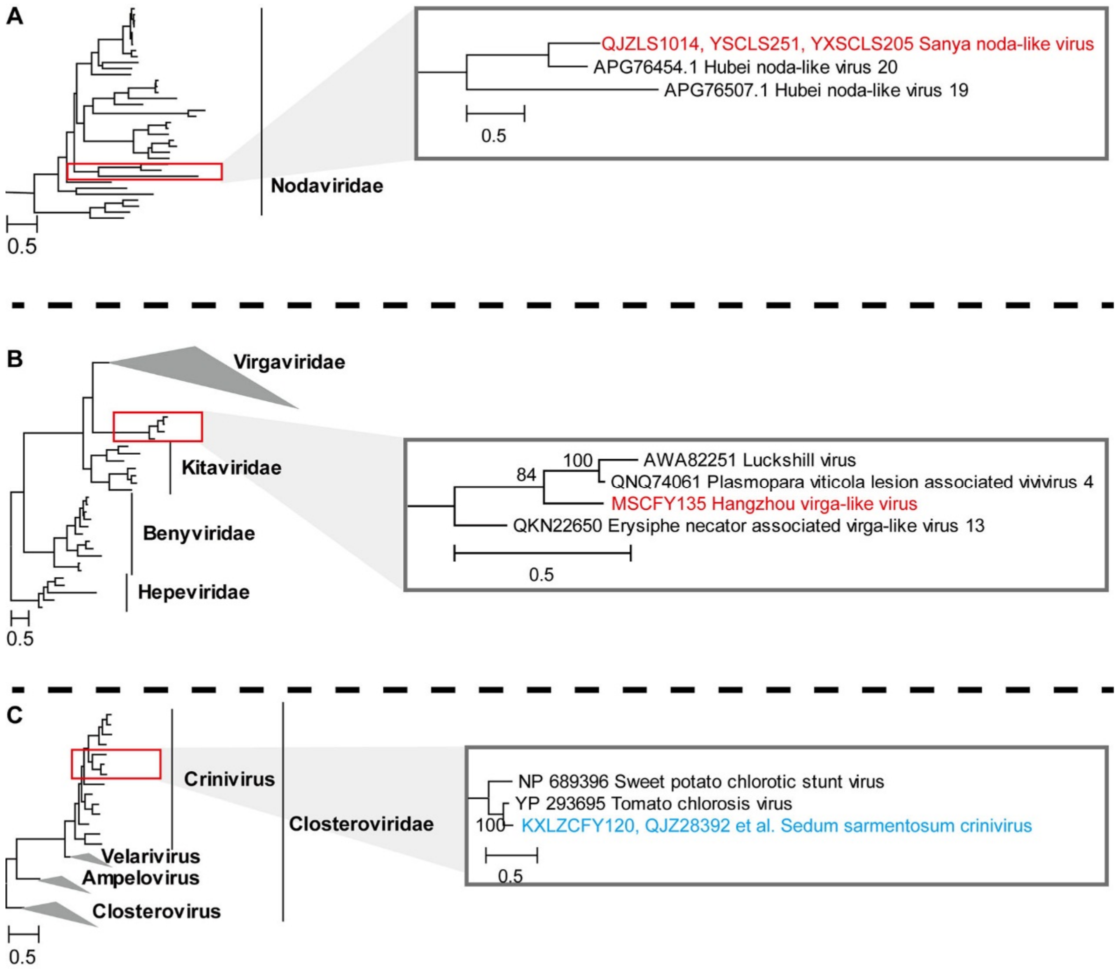

Figure S6. ML phylogenetic analysis of the family *Nodaviridae* **(A)**, *Virgaviridae* **(B)** and *Closteroviridae* **(C)**. Phylogenetic trees were constructed using the aligned sequences of RdRp. The names of new viruses discovered in this study are marked in red, the names of known viruses discovered in this study are marked in blue, the reference virus serial numbers and names are marked in black. The genera of viruses (and branches in minimized trees) in this study are marked in bold.

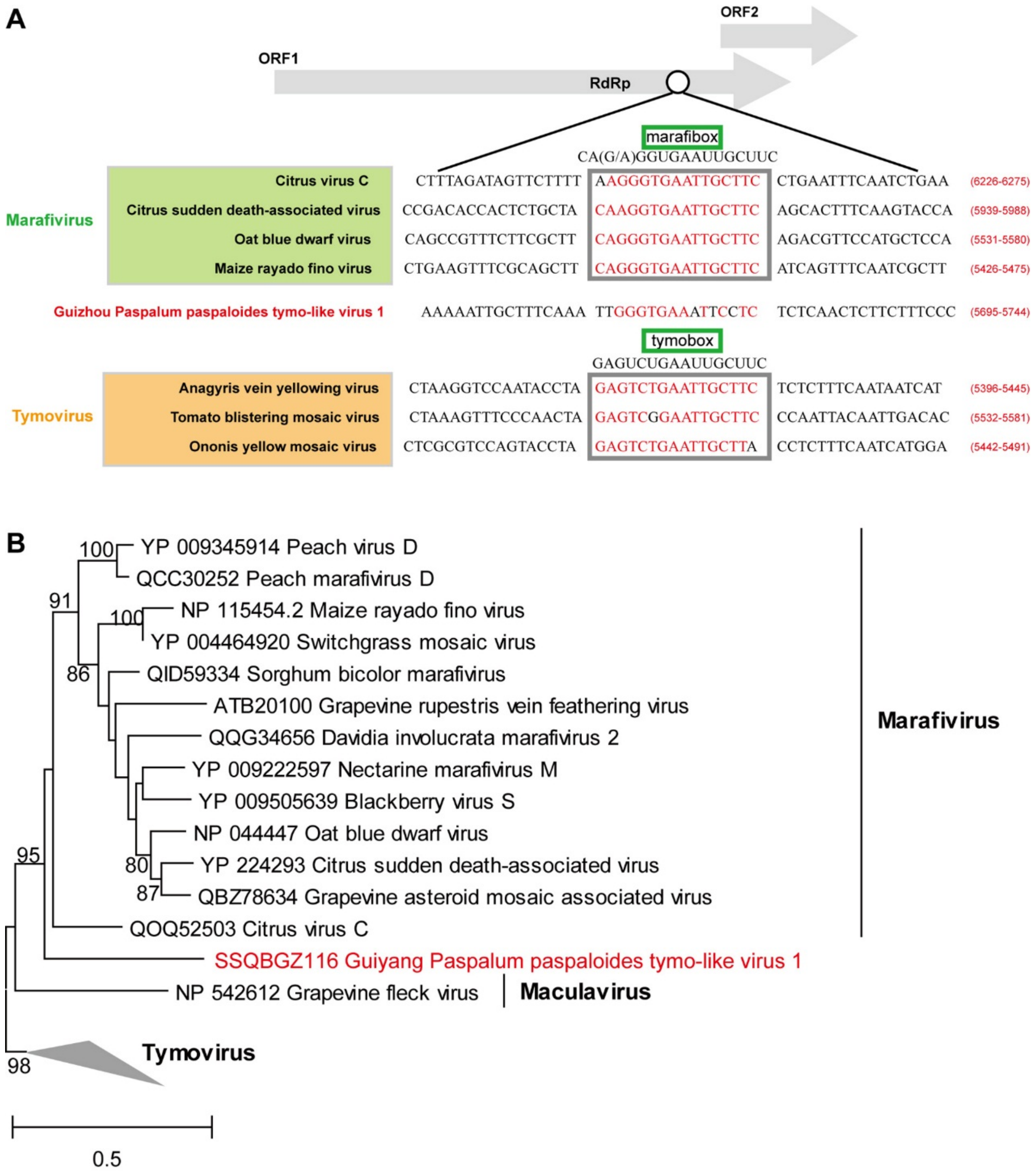

Figure S7. The conserved sequence and ML phylogenetic analysis of the family *Tymoviridae*. **(A)** The 16-nt conserved sequence at the ORF1 3' end of Guiyang Paspalum paspaloides tymo-like virus 1 (GPTV1). The conserved region is marked in the box, the sequence name is shown on the left side of viral sequence, the genus to which the virus belongs is marked on the left side of the virus name, and the location of the genome where the sequence is located is on the right side of the sequence. **(B)** ML phylogenetic analysis of the family Tymoviridae. Phylogenetic trees were constructed using the aligned sequences of RdRp. The names of new viruses discovered in this study are marked in red, the reference virus serial numbers and names are marked in black. The genera of viruses (and branches in minimized trees) in this study are marked in bold.

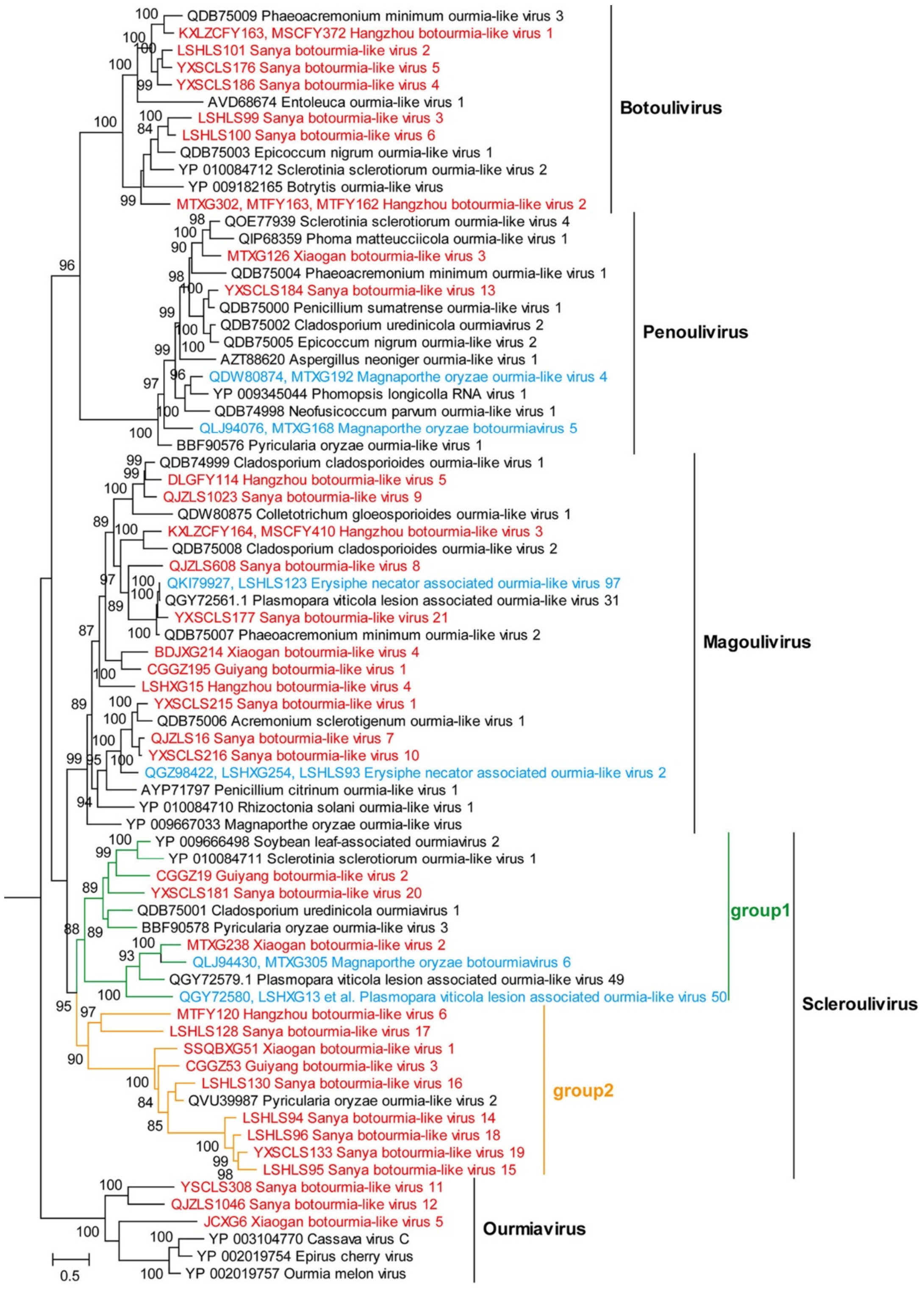

Figure S8. ML phylogenetic analysis of the family *Botourmiaviridae*. Phylogenetic trees were constructed using the aligned sequences of RdRp. The names of new viruses discovered in this study are marked in red, the names of known viruses discovered in this study are marked in blue, the reference virus serial numbers and names are marked in black. The genera of viruses (and branches in minimized trees) in this study are marked in bold.

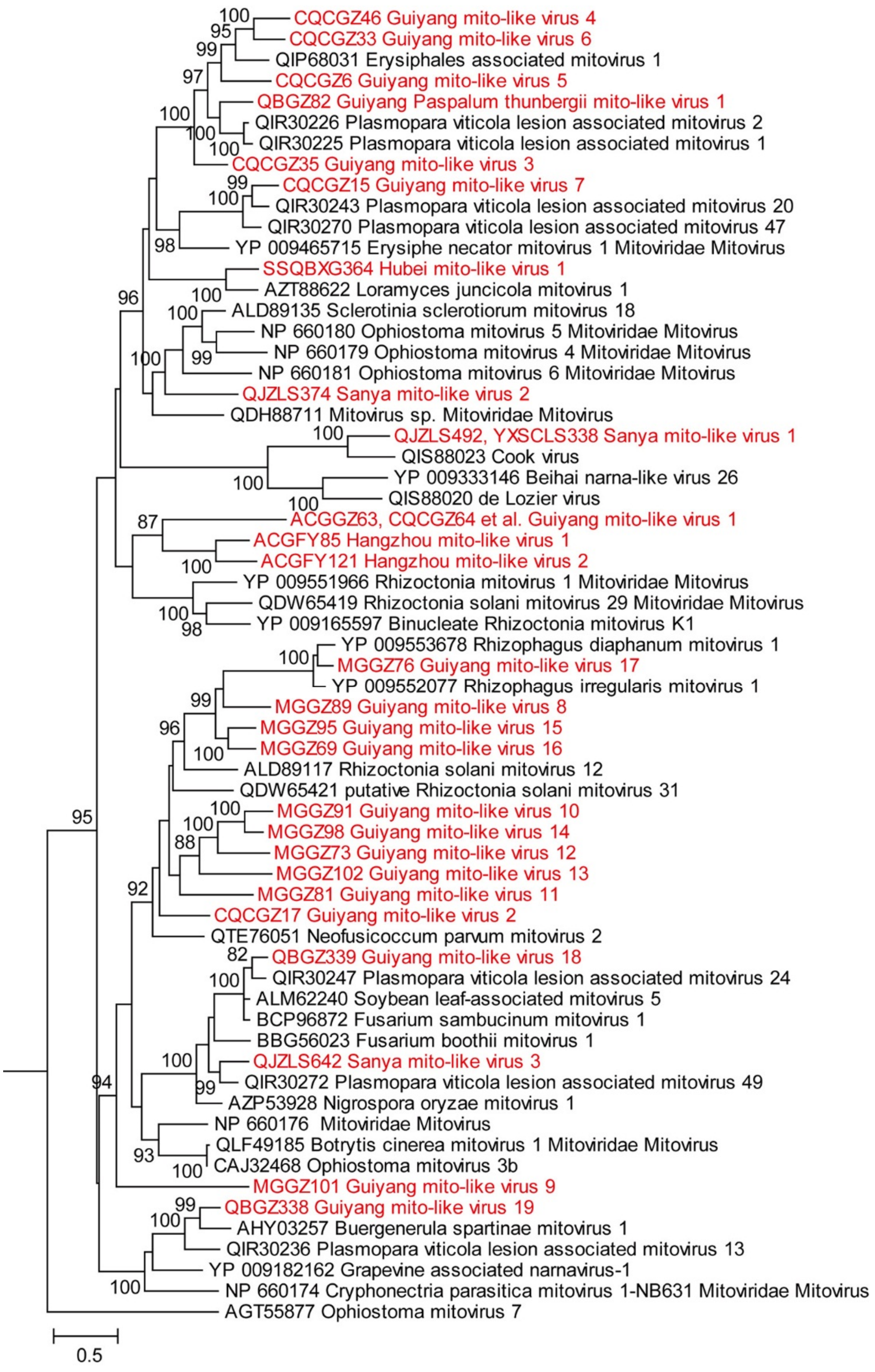

Figure S9. ML phylogenetic analysis of the family *Mitoviridae*. Phylogenetic trees were constructed using the aligned sequences of RdRp. The names of new viruses discovered in this study are marked in red, the reference virus serial numbers and names are marked in black. The genera of viruses (and branches in minimized trees) in this study are marked in bold.

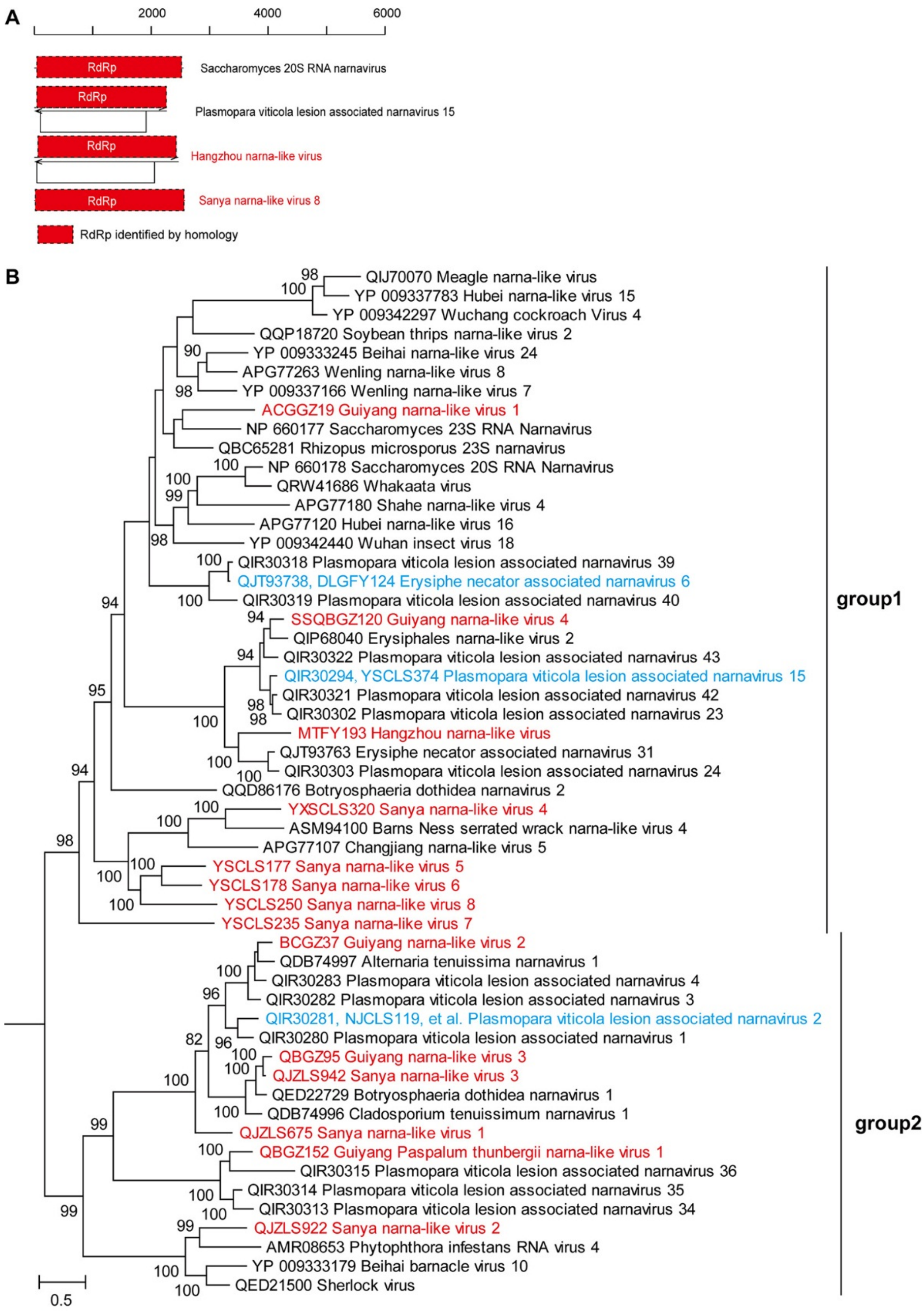

Figure S10. Representative genome organizations and ML phylogenetic analysis of the family *Narnaviridae*. **(A)** The predicted ORF is represented by a rectangular box, and the protein domain encoded by predicted ORF is marked in boxes. The names of new viruses in this study are marked in red; the names of the reference viruses are marked in black. **(B)** Phylogenetic trees were constructed using the aligned sequences of RdRp. The names of new viruses discovered in this study are marked in red, the reference virus serial numbers and names are marked in black. The genera of viruses (and branches in minimized trees) in this study are marked in bold.

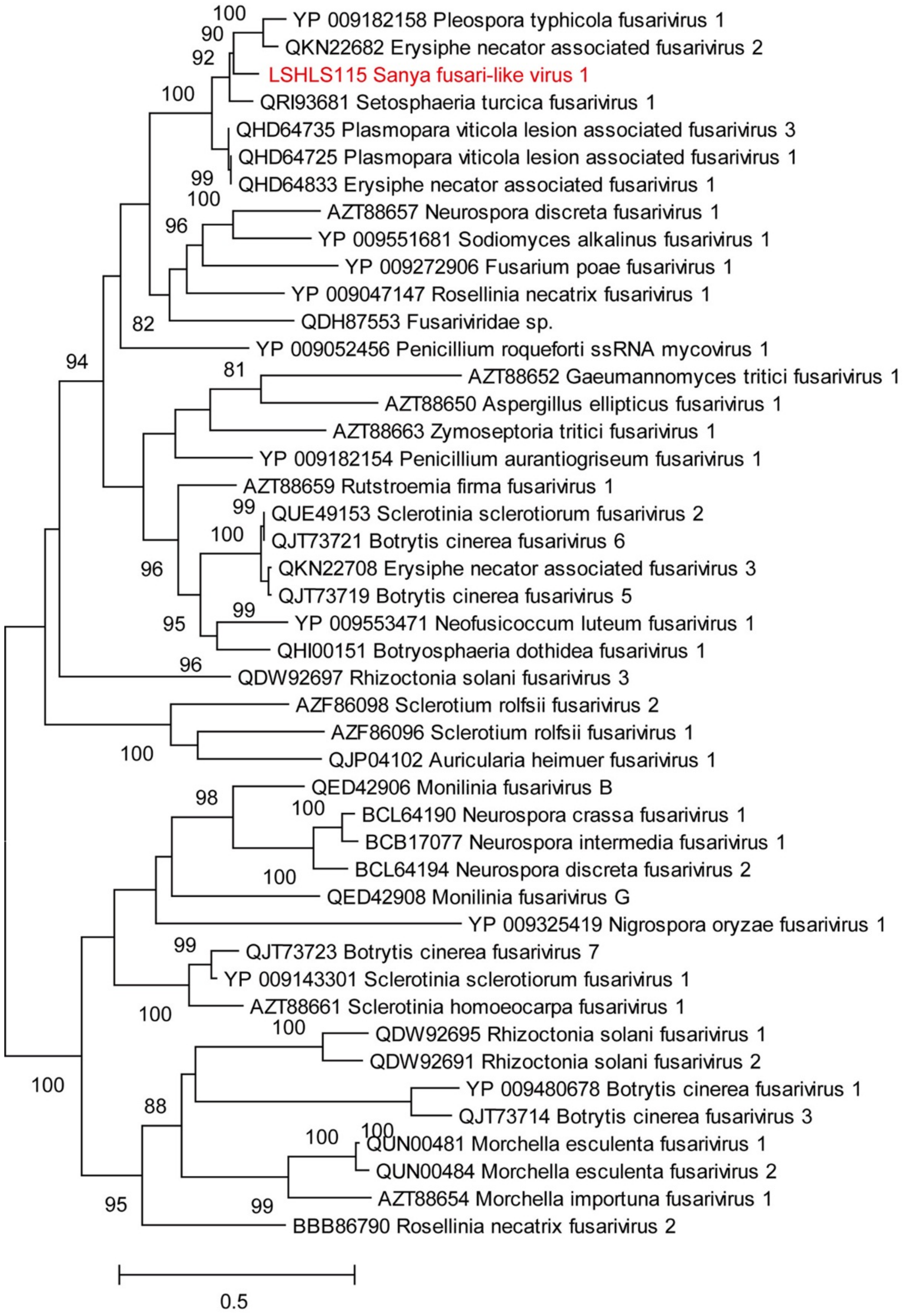

Figure S11. ML phylogenetic analysis of the family *Fusariviridae*. Phylogenetic trees were constructed using the aligned sequences of RdRp. The names of new viruses discovered in this study are marked in red, the reference virus serial numbers and names are marked in black. The genera of viruses (and branches in minimized trees) in this study are marked in bold.

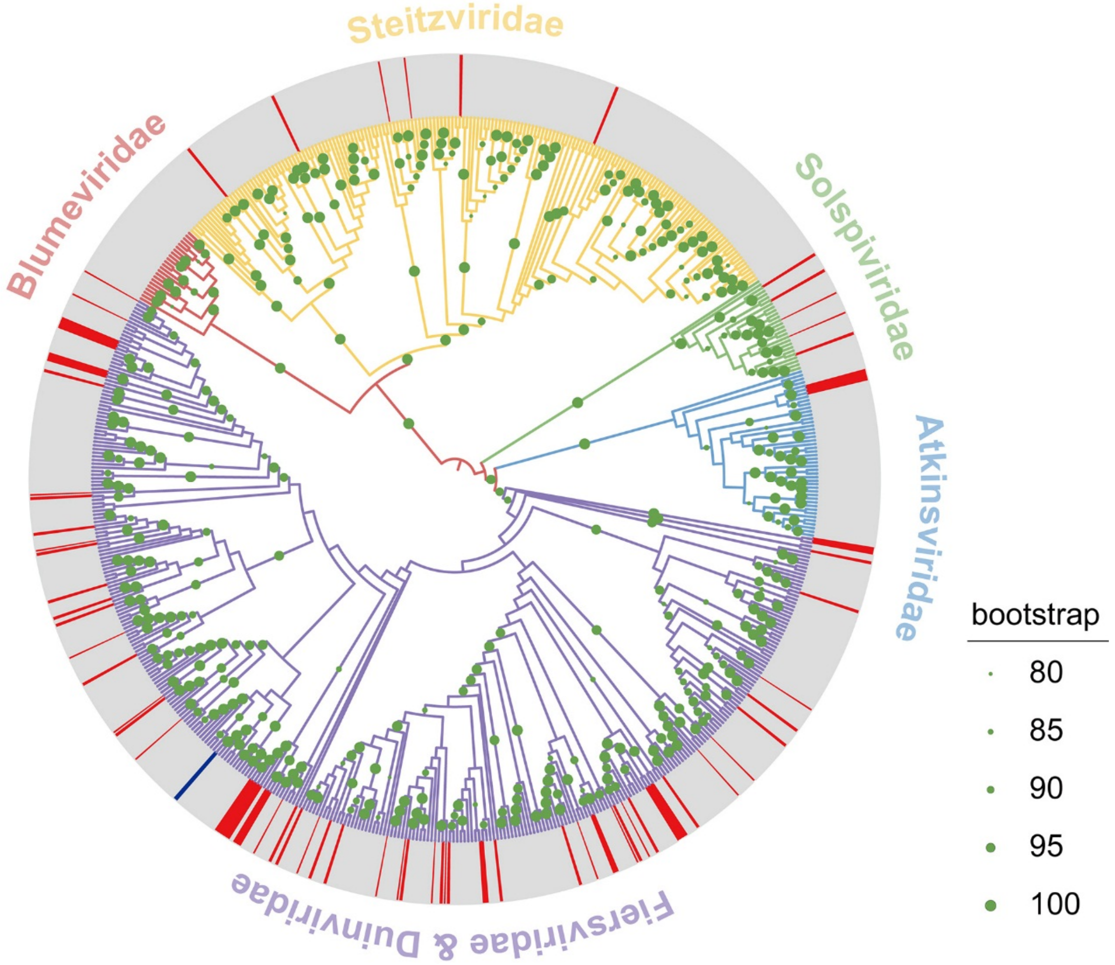

Figure S12. A fully labelled ML phylogeny for the class *Leviviricetes*. A phylogenetic tree was constructed using the aligned sequences of RdRp. The new viruses discovered in this study are shaded red, the known viruses discovered in this study are shaded blue, and the reference viruses described previously are shaded gray. The families for viruses in this study are shown in bold; the color of the clade matches the color of the family name.

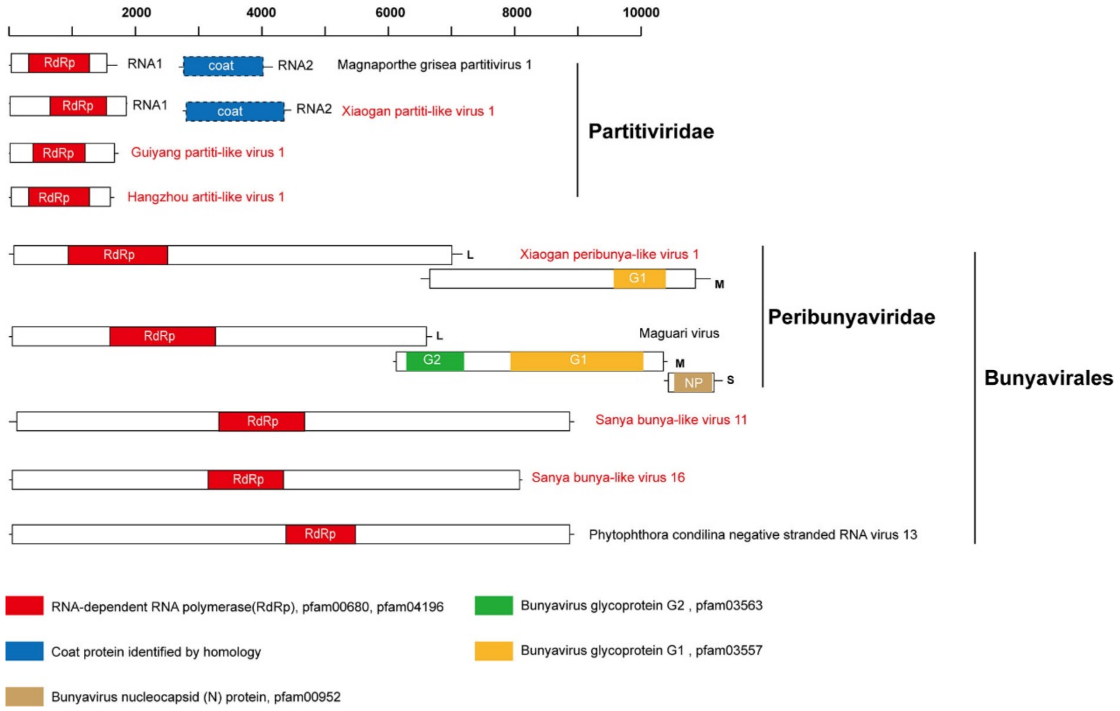

Figure S13. Representative genome structure of viruses classified into the family *Partitiviridae* and the order *Bunyavirales*. The predicted ORF is represented by a rectangular box, and putative protein domain encoded by the predicted ORF is marked in red in the boxes. The names of new viruses in this study are marked in red; the names of the closest relatives are marked in black. The families of viruses are classified and marked on the right side of the genome structure.
